# Rhinitis Symptoms and Asthma among Parents of Preschool Children in Relation to the Home Environment in Chongqing, China

**DOI:** 10.1371/journal.pone.0094731

**Published:** 2014-04-14

**Authors:** Juan Wang, Baizhan Li, Wei Yu, Qin Yang, Han Wang, Duchai Huang, Jan Sundell, Dan Norbäck

**Affiliations:** 1 Department of Medical Sciences, Uppsala University and University Hospital, Uppsala, Sweden; 2 Key Laboratory of Three Gorges Reservoir Region's Eco-Environment, Ministry of Education, Chongqing University, Chongqing, China; 3 Department of Building Science, Tsinghua University, Beijing, China; Kliniken der Stadt Köln gGmbH, Germany

## Abstract

Risk factors for rhinitis and asthma in the home environment were studied by a questionnaire survey. Parents of 4530 1–8 year old children (one parent per child) from randomly selected kindergartens in Chongqing, China participated. 70.4% were females; 47.1% had rhinitis symptoms in the last three months (current rhinitis, CR); 1.6% reported a history of allergic asthma (AA); 2.7% reported a history of allergic rhinitis (AR); 16.4% were current smokers; 50.8% males and 2.4% females were current smokers. Stuffy odor, unpleasant odor, tobacco smoke odor and dry air were associated with CR (adjustment for gender, current smoking and other perceptions of odor or humidity). Associations between home environment and CR, AR, and AA were studied by multiple logistic regression analyses, adjusting for gender, current smoking and other significant home factors. Living near a main road or highway was a risk factor for both CR (OR(95%CI): 1.31(1.13,1.52)) and AR (OR(95%CI): 2.44(1.48,4.03)). Other risk factors for CR included living in rural areas (OR(95%CI): 1.43(1.10,1.85)), new furniture (OR(95%CI): 1.28(1.11,1.49)), water damage (OR(95%CI): 1.68(1.29,2.18)), cockroaches (OR(95%CI): 1.46(1.23,1.73)), and keeping pets (OR(95%CI): 1.24(1.04,1.49)). Other risk factors for AR included redecoration (OR(95%CI): 2.14(1.34,3.41)), mold spots (OR(95%CI): 2.23(1.06,4.68)), window pane condensation (OR(95%CI): 2.04(1.28,3.26)). Water damage was the only home factor associated with AA (2.56(1.34,4.86)). Frequently put bedding to sunshine was protective for CR (OR(95%CI): 0.79(0.68,0.92); cleaning every day was protective for AR (OR(95%CI): 0.40(0.22,0.71)). In conclusion, parents' CR and AR were related to a number of factors of the home environment.

## Introduction

Rhinitis is a worldwide health problem that generates a significant healthcare burden among people in all ages. Although it is not a life threatening condition, its negative impact on human's quality of life has been reported in studies from different countries [1]–[4].

Rhinitis can be divided into allergic rhinitis (AR) and non-allergic rhinitis (NAR). It is considered to be allergic rhinitis (AR) when these symptoms are accompanied by allergen-specific IgE production. The absence of outdoor symptoms in the spring, no parental history of allergy, no symptoms around cats, the presence of symptoms around perfumes and fragrances, are predictive of NAR [5]. Pollen, allergens from certain pets, house dust mites and molds are common causes of allergic rhinitis. Moreover, indoor air pollution, lifestyle factors (ownership of pets, infant feeding, childhood infections, nutrient intake, family and sibship size, hygiene standard, food, vaccination, stress) and socioeconomic conditions, are quoted as adjuvant factors for allergic sensitization and possible causes of the increased prevalence [6]. NAR is when obstruction and rhinorrhea occurs in relation to non-allergic, noninfectious triggers such as change in the weather, exposure to caustic odors or cigarette smoke, barometric pressure differences, etc [7].

Females [8], [9], young adults [9]–[11], and those with allergic heredity [12] are at higher risk of having AR. Home exposures may contribute to the development of AR. Allergen sources from home, such as having pets [13] and the presence of cockroaches [11], [14] are important risk factors for AR. Chinese studies has shown that house dust mite allergy was the most common allergy in AR patients [15], [16]. Building dampness can be another risk factor for AR [17], [18]. Few studies have reported associations between levels of microbiologic agents and AR [19]. Cooking fumes exposure [11] and using wood as heating and wood or biomass as cooking material are other risk factors for AR [17]. Moreover, environment tobacco smoke (ETS) can induce nasal obstruction and decreased muco-ciliary clearance in NAR [20], [21].

During past decades, the prevalence of allergic rhinitis has increased, especially in developed countries. The prevalence of adults' rhinitis seems to still be increasing but the prevalence is different in different countries [22], [23]. The increased diagnostic activity and observance may have contributed to the increase. AR is a common manifestation of allergic disease affecting approximately 10%–25% of the world population [24], and has been reported to be one of the top ten reasons for visits to primary care clinics [25]. One cross-sectional study in Turkey in parents of primary school children showed that the prevalence of AR was: 17.5% for males and 21.2% for females in rural areas; 11.7% for males and 17.0% for females in urban areas [17]. The prevalence of AR increased from 22% to 31% from 1990 to 2008 in Swedish young adults [26]. Although the prevalence and epidemiologic features of AR are well defined, less information is available on the prevalence of NAR in the general population. It is estimated that NAR affects as many as 17 million Americans [27]. One study on 975 patients reported that 43% of them had only AR, 23% had only NAR, and 34% had mixed rhinitis [27]. Many people with rhinitis have both AR and NAR, which makes it difficult to study pure AR or NAR [28]. One study has shown that a substantial subset of individuals with AR were in addition hyperreactive to non-allergic triggers [29].

Rhinitis and asthma commonly coexist. A recent study found that 64% of asthmatic patients had AR and 20% of AR patients had asthma [30]. Another study showed that 60% of asthmatics reported AR [31]. Rhinitis is a significant risk factor for asthma incidence [32]. Not only the intensity, but also the duration of rhinitis is correlated with the development of asthma [33]. A survey in Chinese patients with asthma and/or rhinitis found that patients with both asthma and rhinitis had higher prevalence of positive skin-prick tests (SPT) and specific IgE positivity to most allergens as compared to those with asthma or rhinitis alone [34]. Rhinitis and asthma may be manifestations of the same disease and share common pathologic and physiologic characteristics, as proposed by the 'united airways' hypothesis [35].

There is a lack of epidemiological studies on environmental risk factors for rhinitis in China, and we have found no previous study about the home environment and rhinitis in Chinese adults. In this cross-sectional study the main aim is to estimate cumulative incidence of allergic asthma and allergic rhinitis, and prevalence of current rhinitis in Chongqing adults with young children, and to identify domestic environmental factors associated with these health variables. In addition, we studied the association between perceptions of odors and air humidity and rhinitis and asthma.

## Methods

### Ethics Statement

The study and the consent procedure were approved by the Medical Research Ethics Committee of School of Public Health, Fudan University. The participants gave informed consent.

### Selection of the study subjects

The present study is a part of an epidemiological multi-center study of asthma and allergies among children and their relation to the home environment in China (China, Children, Homes,Health, CCHH) [36]–[38]. The study is using the same study protocol and questionnaire as earlier studies [39], [40], starting with a cross-sectional questionnaire survey followed by a nested case-control study. The survey was carried out from December 2010 to April 2011.

The questionnaires were distributed to children's parents through teachers in kindergartens in three districts (Shapingba, Jiulongpo, Yubei) that were randomly selected from 9 districts of Chongqing city. From the 54 randomly selected kindergartens (15 from Shapingba, 21 from Jiulongpo and 18 from Yubei), 7117 subjects (one parent per child aged from 1–8 years old) were selected and invited for the questionnaire survey. Completed questionnaires were collected one week later by the teachers.

### Questionnaire

A modified version of a self-administered questionnaire previously used in Sweden, and among Chinese university students [39], [40] has been used in this study. The questionnaire was slightly modified to be more appropriate for Chinese culture, lifestyle, building structure and interior characteristics.

Questions on subjects' gender, own current smoking habit, current smoking of the spouse, rhinitis symptoms in the last three months (current rhinitis), history of allergic rhinitis (cumulative incidence of allergic rhinitis), history of allergic asthma (cumulative incidence of allergic asthma), and perceptions of odors and air humidity were collected by a self-reported questionnaire.

Question about current rhinitis followed the Northern Swedish Office Illness study [41]. “During the last three months, have you had irritating, stuffy or runny nose?” There were three options: often (every week); sometimes; never. Any current rhinitis was defined as those who reported often or sometimes; weekly current rhinitis was defined as those reported often; less common current rhinitis was defined as those who reported sometimes.

Questions about odor and air humidity perceptions were: during the last three months, have you had any of the following perceptions: Stuffy odor; Unpleasant odor; Pungent odor; Mold odor; Tobacco smoke odor; Humid air; Dry air. There were three options: weekly; sometimes; never. For each odor or humidity perception, “Yes” was defined as those who reported weekly or sometimes; “No” was defined as those who only reported never.

Questions about exposure indicators and building characteristics contained in present paper are:

1. House site (urban/suburban/rural);
* Whether current residence is near a main road or highway with a distance of 200 m (yes/no);
3. Building construction year (before 1980/1980–1990/1991–2000/2001–2005/after 2005);
4. Residence area (< = 40 m^2^/41–60 m^2^/61–75 m^2^/76–100 m^2^/101–150 m^2^/>150 m^2^);
5. Wall materials on children's bedroom (wall paper/cement/lime/paint/emulsion paint/other);
6. Floor materials (wood/cement/ceramic tile or stone/laminated floor/other);
7. Whether any redecoration has been done since one year before pregnancy (yes/no);
* Whether any new furniture has been bought since one year before pregnancy (yes/no);
* Whether subject has reported any mold spots in child bedroom (yes/no);
* Whether subject has reported any damp stains in child bedroom (yes/no);
* Whether subject has reported any water damage in child bedroom (yes/no);
* Whether subject has reported window pane condensation during winter in child bedroom (yes/no);
* Whether subject has seen cockroaches in home before (yes/no);
* Whether subject has seen rats in home before (yes/no);
* Whether subject has seen mosquitoes/flies in home before (yes/no);
* Whether subject has used mosquito-repellent incense in home before (yes/no);
* Whether subject has used incense in home before (yes/no);
* Whether subject has pets in home currently (yes/no); if yes, it is (cat/dog/rodent (rabbit/rat)/bird/aquarium fishes or reptiles/other);
19. The frequency of cleaning child's bedroom (every day/Less or equal twice a week);
* The frequency of putting child's bedding to sunshine (frequently/never or rarely);
21. The frequency of opening window in child's bedroom in winter (frequently/never or rarely).

“*” included in a risk factor score.

### Indoor environment nasal risk factors score

From twenty one questions about exposure indicators and building characteristics above, an indoor environment risk factors score (0–13) was constructed (Continuous Score, range from 0–13), by adding the number of yes response to 13 exposure indicators of home environment significantly associated with current rhinitis (which marked with “*”, question 20 was changed by asking if put bedding to sunshine frequently (yes/no), “yes” was coded as “0” and “no” was coded as “1”).

The Continuous Score was then classified in five categories to make another Categorized Score: by adding number of indoor environment risk factors, separately as score category 0 (0, 1, 2, 3 and 4 risk factors out of 13), score category 1 (5 risk factors out of 13), score category 2 (6 out of 13), score category 3 (7 out of 13) and score category 4 (8 or more out of 13).

From four questions about indoor dampness above, an indoor dampness score (0–4) was constructed (Continuous Dampness Score, range from 0–4), by adding the number of yes response to four dampness indicators of home environment (question 9–12). The Continuous Dampness Score was then classified in three categories to make another Categorized Dampness Score: by adding different numbers dampness indicators, separately as score category 0 (0 dampness indicators out of 4), score category 1 (1 dampness indicator out of 4), score category 2 (2 or more out of 4).

### Statistical analysis

All statistical analyses were conducted with SPSS 17.0. Initially, factor analysis was applied to all exposure indicators and building characteristics questions except question 3 which is about building construction year (question 5 was changed to five separate questions by given yes and no answers to each type of wall material; question 6 was changed to four separate questions by given yes and no answers to each type of floor material). Principal component analysis and rotated component matrix (varimax with Kaiser normalisation) was used.

Then, the association between gender and current rhinitis, history of allergic rhinitis, history of allergic asthma were analyzed by multiple logistic regression with adjustment of current smoking. The association between current smoking and current rhinitis, history of allergic rhinitis, history of allergic asthma were analyzed by multiple logistic regression with adjustment of gender.

Initially, associations between building characteristics and exposure indicators and current rhinitis, history of allergic rhinitis, history of allergic asthma were evaluated in multiple logistic regression by analyzing one factor each time with adjustment of gender and current smoking.

Since the number of variables analyzed in the models was relatively large, stepwise multiple logistic regression models (forward elimination, condition method) were used to find the most significant variables for current rhinitis, history of allergic rhinitis and history of allergic asthma. Then, reduced multiple logistic regression models were applied by adding gender, current smoking and all significant factors from stepwise regression models. Multi-nominal regression models were applied for weekly and less common current rhinitis by adding the same factors as in the reduced multiple logistic regression models. Results achieved by the reduced multiple models and multi-nominal models were compared with initial logistic regression models.

As a next step, associations between current rhinitis and Continuous Score, Categorized Score (score 0–4), Categorized Dampness Score were calculated by multiple logistic regression adjusting for gender and current smoking. Odds Ratios (OR) for Continuous Score were calculated by one unit increase on the 13 steps.

Associations between each odor and humidity perception and current rhinitis, history of allergic rhinitis, history of allergic asthma were calculated by multiple logistic regression with mutual adjustment including gender, current smoking and other six types of odors or humidity perceptions.

Percentages given for each question are for valid data. The reference category for logistic and multi-nominal regression were subjects never having the particular symptom. Associations were expressed as odds ratios (OR) with a 95% confidence interval (CI) for logistic regression but relative risk ratios (RRR) with a 95% confidence interval (CI) for multi-nominal regression. Analyses were considered to be statistically significant if the *p*-value is less than 0.05. In all statistical analysis, two-tailed tests and a 5% level of significance were applied.

## Results

Totally, 5299 parents out of 7117 who were invited to answer the questionnaire (one parent per child) participated, total response rate is 74.5%. The fluctuation of different kindergartens' response rate was small. In this analysis, 4530 complete questionnaires answered by children' parents were included, excluding 769 questionnaires answered by children's grandparents or other persons.

The prevalence of history of allergic asthma, history of allergic rhinitis, current rhinitis and current smoking is shown in Table 1. In total, missing values for history of allergic asthma, history of allergic rhinitis, current rhinitis and current smoking and were 311, 311, 564 and 222, respectively. Men had less history of allergic rhinitis and were more often current smokers. Among those with history of allergic rhinitis, 12.1% did not have current rhinitis, 60.7% had less common current rhinitis and 27.1% had weekly current rhinitis. Among those without any current rhinitis, 0.6% reported history of allergic rhinitis; among those with less common current rhinitis, 3.9% reported history of allergic rhinitis and among those with weekly current rhinitis, 24.8% reported history of allergic rhinitis.

**Table 1:** The prevalence of history of allergic asthma, history of allergic rhinitis, current rhinitis and current smokers among participating parents (%) (n = 4530).

Demographic information		Total	Male	Female	*p*-value
		100	29.6	70.4	
History of allergic asthma		1.6	1.6	1.6	0.934^a^
History of allergic rhinitis		2.7	1.6	3.2	0.005^a^
Current rhinitis	Weekly	3.1	2.9	3.2	
	Less common	44.0	43.0	44.5	
	Never	52.9	54.1	52.3	0.533^b^
Current smoker^c^		16.4	50.8	2.4	<0.001^a^

a *p*-value Chi-square test for 2×2 contingency table.

b *p*-value Chi-square test for 2×3 contingency table.

c Subjects who are current smokers.

Perceptions of odor and humidity were risk factors for rhinitis but not for asthma. Tobacco smoke odor was a risk factor for current rhinitis and history of allergic rhinitis in logistic regression with mutual adjustment of gender, current smoking and other odors and humidity perceptions. Stuffy odor, unpleasant odor and the perception of dry air were all associated with current rhinitis in logistic regression with mutual adjustment of other odors and humidity perceptions, gender and current smoking (Table 2).

**Table 2:** Associations between current rhinitis, history of allergic rhinitis, history of allergic asthma and odors and humidity perceptions OR(95%CI)^a^.

Type of odor	Current rhinitis	*p*-value	History of allergic rhinitis	*p*-value	History of allergic asthma	*p*-value
Stuffy odor	1.51(1.28,1.79)	<0.001	1.12(0.69,1.80)	0.653	0.78(0.41,1.51)	0.465
Unpleasant odor	1.41(1.17,1.69)	<0.001	1.38(0.84,2.27)	0.209	1.83(0.96,3.51)	0.069
Pungent odor	1.18(0.96,1.45)	0.125	1.37(0.81,2.34)	0.241	0.70(0.31,1.56)	0.378
Mold odor	1.02(0.80,1.30)	0.876	0.49(0.22,1.07)	0.072	1.53(0.65,3.60)	0.327
Tobacco smoke odor	1.38(1.19,1.61)	<0.001	1.73(1.12,2.69)	0.014	1.68(0.94,3.00)	0.081
Humid air	1.17(0.99,1.38)	0.061	0.94(0.59,1.52)	0.813	0.57(0.29,1.13)	0.107
Dry air	1.27(1.09,1.49)	0.003	1.58(1.004,2.50)	0.048	1.60(0.88,2.89)	0.121

a Calculated by logistic regression with mutual adjustment, odds ratios were adjusted for gender and current smoking.

The prevalence of home environmental characteristics is shown in Table 3. The majority of the homes were from urban areas. Subjects with current rhinitis associated with more report of home risk factors than others.

**Table 3:** Home environmental characteristics of participating parents (n = 4530).

		Current rhinitis		Total
		Yes(%)	No(%)	%
House site	Urban	70.4	72.4	71.3
	Suburban	18.0	19.0	18.6
	Rural	11.6	8.6*	10.1
Living near a main road or highway^a^	Yes	48.3	40.5***	44.2
Construction year	Before 1980	3.5	3.6	3.6
	1980–1990	9.5	8.4	9.1
	1991–2000	22.6	21.7	22.6
	2001–2005	35.1	35.3	34.7
	After 2005	29.2	31.0	29.9
Area	< = 40 m^2^	14.8	13.4	14.4
	41–60 m^2^	13.0	13.4	13.3
	61–75 m^2^	18.5	18.2	18.4
	76–100 m^2^	26.0	26.4	26.1
	101–150 m^2^	22.6	23.6	22.9
	>150 m^2^	5.1	4.9	4.9
Wall material	Wall paper	11.8	11.9	12.0
	Paint	8.7	8.9	8.8
	Lime	19.5	18.3	19.0
	Cement	8.9	7.1	8.1
	Emulsion paint	46.2	49.1	47.1
	other	8.0	6.1	7.5
Floor material	Wood floor	15.4	19.5	17.7
	Cement	19.2	15.2	17.5
	Ceramic tile/Stone	35.0	33.3	34.2
	Laminated	28.5	29.0	28.1
	Other	2.0	2.2***	2.2
Redecoration^b^	Yes	34.8	33.0	34.1
New furniture^c^	Yes	60.4	54.3***	57.4
Dampness^d^	Yes	53.0	42.8***	47.3
Mold spots	Yes	6.9	4.0***	5.4
Damp stains	Yes	10.6	6.5***	8.4
Water damage	Yes	12.0	6.5***	9.2
Window pane condensation	Yes	36.7	31.9**	33.5
Cockroaches^e^	Yes	79.4	71.6***	76.1
Rats^e^	Yes	46.1	40.6**	44.3
Mosquitoes/flies^e^	Yes	88.6	82.5***	85.5
Current pets	Yes	22.8	18.5**	20.8
Mosquito-repellent incense^f^	Yes	88.7	85.1**	86.7
Incense^f^	Yes	19.4	15.3**	17.1
Cleaning every day	Yes	39.4	41.9	41.1
Frequently put bedding to sunshine	Yes	37.5	43.5**	40.6
Frequently open window in winter	Yes	34.0	36.0	35.7

a Subject's home located within a distance of 200 meters of a main road or highway.

b Subject's home has been redecorated/renovated since 1 year before pregnancy.

c Subject's home has acquired new furniture since 1 year before pregnancy.

d Subject has reported any of the four dampness signs at home: mold spots, damp stains, water damage or window pane condensation during winter in child's bedroom.

e Subject has seen cockroaches/rats/mosquitoes/flies in home.

f Subject has used mosquito-repellent incense/incense in home.

*p<0.05;

**p<0.01;

***p<0.001.

There was no significant association between gender and current rhinitis, history of allergic rhinitis, history of allergic asthma in logistic regression models with adjustment of current smoking. Current smoking was not associated with current rhinitis, history of allergic rhinitis in logistic regression with adjustment of gender, but current smoking was negatively associated with history of allergic asthma (OR(95%CI): 0.39(0.15,0.99), P = 0.047). Spouses' current smoking (passive smoking) was a risk factor for non-smoking females' current rhinitis (OR(95%CI): 1.17(1.004,1.36), P = 0.044), however, no association was found between passive smoking among non-smoking females and history of allergic rhinitis, history of allergic asthma. When adjusting for gender and current smoking, history of allergic asthma was associated with current rhinitis and history of allergic rhinitis (Table 4); history of allergic rhinitis was associated with current rhinitis (OR(95%CI): 9.09(4.96,16.7), P<0.001).

**Table 4:** Associations between house site, construction year, area, history of allergic asthma and current rhinitis, history of allergic rhinitis calculated by logistic regression OR(95%CI)^a^.

		Current rhinitis	*p*-value	History of allergic rhinitis	*p*-value
House site	Urban	1.00		1.00	
	Suburban	1.00(0.85,1.18)	0.991	0.47(0.25,0.87)	0.017
	Rural	1.36(1.10,1.69)	0.005	0.38(0.15,0.94)	0.037
Construction year	After 2001	1.00		1.00	
	Before 2000	1.08(0.94,1.23)	0.274	0.75(0.50,1.14)	0.183
Area	>75 m^2^	1.00		1.00	
	< = 75 m^2^	1.05(0.92,1.19)	0.452	0.69(0.46,1.02)	0.062
Cumulative incidence of allergic asthma		3.40(1.89,6.14)	<0.001	7.51(3.81,14.8)	<0.001

a Odds ratios were adjusted for gender and current smoking.

Factor analysis was applied to identify environmental characteristics patterns by including all questions except building construction year. Question 5 was changed to five separate questions by given yes and no answers to each type of wall material, and question 6 was changed to 4 separate questions by given yes and no answers to each type of floor material. Out of a total of 27 variables, eleven different factors were identified:

1. area, lime wall and cement floor (area > = 75 m^2^ was associated with lime wall and cement floor);
2. house site (urban/suburban/rural), cement wall and cement floor (there was a trend of increasing presence of cement walls and cement floors from urban to suburban and rural areas);
3. wall paper and wood floor;
4. ceramic/stone floor and laminated floor (negative association);
5. paint and emulsion paint (negative association);
6. redecoration and new furniture;
7. mold spots and damp stains;
8. window pane condensation and frequently open window in winter (negative association).
9. cockroaches and rats;
10. mosquitoes/flies and mosquito repellent incense;
11. cleaning every day and frequently put bedding to sunshine;

Four variables were not included in any factor: living near a main road or highway, water damage, current pets and use of incense.

Associations between house site, construction year, area, history of allergic asthma, and current rhinitis, history of allergic rhinitis calculated by logistic regression adjusting for gender and current smoking, are shown in Table 4. Subjects living in rural areas had more current rhinitis, but those living in suburban and rural areas had less history of allergic rhinitis.

Associations between home environmental characteristics and current rhinitis, history of allergic rhinitis calculated by logistic regression adjusting for gender and smoking are shown in Table 5. Living near a main road or highway, new furniture, having pets and indicators of an impaired indoor environment, such as with dampness problems, cockroaches, rats, mosquitoes/flies, current pets, using mosquito-repellent incense and incense, were all risk factors for current rhinitis. For history of allergic rhinitis, risk factors included living near a main road or highway, redecoration, new furniture, mold spots, window pane condensation and current pets. Compared with ceramic tile/stone floor, those living in homes with wood floor had less current rhinitis. Frequently put bedding to sunshine was protective for current rhinitis. Cleaning every day was protective for history of allergic rhinitis.

**Table 5:** Associations between building characteristics and exposure indicators and current rhinitis, history of allergic rhinitis calculated by logistic regression OR (95%CI)^a^.

		Current rhinitis	*p*-value	History of allergic rhinitis	*p*-value
Living near a main road or highway		1.36(1.20,1.55)	<0.001	2.24(1.50,3.34)	<0.001
Wall materials	Wall paper	1.00		1.00	
	Cement	1.24(0.91,1.68)	0.168	0.36(0.12,1.07)	0.067
	Lime	1.08(0.85,1.37)	0.547	0.48(0.24,0.97)	0.041
	Paint	0.94(0.70,1.26)	0.692	0.85(0.41,1.77)	0.661
	Emulsion paint	0.96(0.78,1.18)	0.677	0.71(0.42,1.21)	0.211
Floor type	Ceramic tile/stone	1.00		1.00	
	Cement	1.20(0.99,1.45)	0.058	0.35(0.15,0.84)	0.018
	Wood floor	0.74(0.62,0.89)	0.002	0.90(0.49,1.60)	0.696
	Laminated floor	0.93(0.80,1.10)	0.406	1.77(1.14,2.73)	0.011
Redecoration		1.09(0.94,1.25)	0.257	2.26(1.51,3.37)	<0.001
New furniture		1.26(1.11,1.44)	<0.001	2.42(1.54,3.81)	<0.001
Mold spots		1.78(1.33,2.39)	<0.001	2.19(1.15,4.16)	0.017
Damp stains		1.67(1.32,2.11)	<0.001	1.62(0.90,2.94)	0.110
Water damage		1.93(1.52,2.44)	<0.001	1.62(0.91,2.88)	0.101
Window pane condensation		1.22(1.06,1.41)	0.006	2.53 (1.69,3.79)	<0.001
Cockroaches		1.54(1.32,1.79)	<0.001	1.47(0.89,2.43)	0.131
Rats		1.26(1.11,1.44)	0.001	0.68(0.45,1.03)	0.068
Mosquitoes/flies		1.62(1.34,1.95)	<0.001	1.90(0.95,3.77)	0.069
Current pets		1.29(1.11,1.51)	0.001	1.80(1.20,2.71)	0.005
Mosquito-repellent incense		1.39(1.15,1.68)	0.001	0.87(0.52,1.47)	0.606
Incense		1.31(1.11,1.56)	0.002	1.54(0.97,2.42)	0.065
Cleaning every day		0.91(0.80,1.03)	0.130	0.44(0.28,0.69)	<0.001
Frequently put bedding to sunshine		0.78(0.69,0.89)	<0.001	0.78(0.52,1.16)	0.220
Frequently open window in winter		0.91(0.79,1.04)	0.154	1.03(0.70,1.53)	0.870

a Odds ratios were adjusted for gender and current smoking habit.

Stepwise logistic regression was applied to reduce the models (Table 5). House site, living near a main road or highway, new furniture, water damage, cockroaches, current pets and frequently open window in winter were significant factors in the stepwise regression for current rhinitis. Reduced multiple logistic and multi-nominal models were applied by adding gender, current smoking and all significant factors from stepwise regression for current rhinitis (Table 6). Living near a main road or highway, redecoration, mold spots, window pane condensation, cleaning every day and frequently open window in winter were significant factors in the stepwise regression for history of allergic rhinitis. Reduced multiple logistic and multi-nominal model was applied for history of allergic rhinitis (Table 7). Water damage was the only significant factor for history of allergic asthma in the stepwise regression. When adjusting for gender and current smoking in reduced multiple models, water damage was still associated with history of allergic asthma (OR(95%CI): 2.56(1.34,4.86), p = 0.004).

**Table 6:** Significant variables identified in reduced multiple models for current rhinitis.

		Any		Less common		Weekly	
		OR(95%CI)^a^	*p*-value	RRR(95%CI)^b^	*p*-value	RRR(95%CI)^b^	*p*-value
Male gender		0.92(0.75,1.12)	0.400	0.92(0.75,1.13)	0.418	0.89(0.49,1.63)	0.714
Current smoker		1.05(0.83,1.34)	0.675	1.04(0.82,1.33)	0.741	1.24(0.61,2.52)	0.559
House site	Urban	1.00		1.00		1.00	
	Suburban	1.13(0.93,1.37)	0.221	1.13(0.93,1.38)	0.225	1.12(0.63,1.98)	0.707
	Rural	1.43(1.10,1.85)	0.007	1.45(1.11,1.88)	0.006	1.10(0.48,2.50)	0.827
Living near a main road or highway		1.31(1.13,1.52)	<0.001	1.29(1.11,1.50)	0.001	1.71(1.10,2.67)	0.018
New furniture		1.28(1.11,1.49)	0.001	1.30(1.12,1.51)	0.001	1.10(0.71,1.72)	0.674
Water damage		1.68(1.29,2.18)	<0.001	1.54(1.18,2.01)	0.002	4.21(2.47,7.16)	<0.001
Cockroaches		1.46(1.23,1.73)	<0.001	1.46(1.22,1.73)	<0.001	1.55(0.89,2.73)	0.125
Current pets		1.24(1.04,1.49)	0.020	1.22(1.01,1.47)	0.036	1.61(0.98,2.65)	0.058
Frequently put bedding to sunshine		0.79(0.68,0.92)	0.002	0.79(0.68,0.92)	0.002	0.74(0.47,1.18)	0.205

a Gender, current smoking and factors significant in stepwise regression (enter model *p*-value level is *p*<0.10).

b Same factors in ^a^ but analyzed by multi-nominal regression.

**Table 7:** Significant variables identified in reduced multiple models for history of allergic rhinitis.

	OR(95%CI)^a^	*p*-value
Male gender	0.66(0.33,1.31)	0.235
Current smoker	0.19(0.04,0.84)	0.029
Living near a main road or highway	2.44(1.48,4.03)	<0.001
Redecoration	2.14(1.34,3.41)	0.001
Mold spots	2.23(1.06,4.68)	0.035
Window pane condensation	2.04(1.28,3.26)	0.003
Cleaning every day	0.40(0.22,0.71)	0.002
Frequently open window in winter	0.84(0.52,1.37)	0.488

a Gender, current smoking and factors significant in stepwise regression (enter model *p*-value level is *p*<0.10).

Logistic regression models for the association between current rhinitis and Continuous Score (range from 0-13) were calculated, adjusted for gender and current smoking. There were significant associations between each unit increase of risk factors and current rhinitis (OR(95%CI): 1.17(1.12,1.23), P<0.001). Logistic regression models for the association between current rhinitis and Categorized Score (0–4) were calculated (Table 8). Results shows that more risk factors were related with higher risk of having current rhinitis, with stratified analysis.

**Table 8:** Associations between current rhinitis and Categorized Score identified in reduced multiple models.

		Any		Less common		Weekly	
Score category 0–4^c^	%	OR(95%CI)^a^	*p*-value	RRR(95%CI)^b^	*p*-value	RRR(95%CI)^b^	*p*-value
Score category 0	33.6	1.00		1.00		1.00	
Score category 1	23.4	1.36(1.10,1.69)	0.005	1.42(1.14,1.77)	0.002	0.54(0.23,1.28)	0.159
Score category 2	20.0	1.62(1.29,2.04)	<0.001	1.65(1.30,2.08)	<0.001	1.33(0.65,2.70)	0.432
Score category 3	14.0	1.69(1.31,2.17)	<0.001	1.65(1.27,2.14)	<0.001	2.15(1.09,4.27)	0.028
Score category 4	9.0	2.17(1.60,2.94)	<0.001	2.10(1.53,2.86)	<0.001	3.15(1.50,6.63)	0.003

a Calculated by multiple logistic regression, odds ratios were adjusted for gender and current smoking.

b Calculated by multi-nominal regression, odds ratios were adjusted for gender and current smoking.

c Categorized Score: by adding numbers of indoor environment risk factors, separately as score category 0 (0, 1, 2, 3 or 4 risk factors out of 13), score category 1 (5 out of 13), score category 2 (6 out of 13), score category 3 (7 out of 13) and score category 4 (8 or more out of 13).

Logistic regression models for the association between current rhinitis and Categorized Dampness Score (0–4) were calculated. 58.7% reported 0 dampness indicators (which is the reference group, score 0); 32.8% reported 1 dampness indicators (score category 1); 8.5% reported 2 or more dampness indicators (score category 2). Compared with score category 0, OR(95%CI) (when adjusting for gender and current smoking) for score category 1 was 1.28(1.09, 1.50) and for score category 2 was 1.91(1.46, 2.49), showed that more dampness indicators were related with higher risk of having current rhinitis.

## Discussions

The prevalence of weekly current rhinitis, history of allergic rhinitis and history of allergic asthma among parents of young children (3–6 years old) in Chongqing were low but current rhinitis occurring less than once a week was common. A number of indoor environmental risk factors were associated with adults' current rhinitis and a history of allergic rhinitis: the most important risk factors were living near a main road or highway, redecoration, new furniture, mold spots, water damage, window pane condensation, the presence of cockroaches, current pets; protective factors were cleaning every day and frequently put bedding to sunshine. Subjects living in rural areas reported more current rhinitis than those in urban areas. Water damage was a risk factor for adults' history of allergic asthma.

Epidemiological studies can be affected by selection bias. In this study, we included all parents from a large cross-sectional study among children in random daycare centers, with no prior information on parents' health status. The sample size was reasonably large, and the response rate was relatively high (74.5%). Thus, a selection bias is fairly unlikely. However, the study population is representative of parents, especially mothers and does not include all ages of adults in Chongqing.

Recall bias is another potential problem. Subjects may overestimate or underestimate their personal symptoms or reports on indoor environment risk factors. Recall bias for rhinitis symptoms should not be a big issue in this study, since questions about rhinitis symptoms are for the last three months. Information bias, in which subjects are aware that certain factors have previously been identified as risks, is another potential problem. However, the risk factors studied in this paper (e.g. wall and floor materials, dampness, and lifestyle), are likely not well known among the Chinese population.

In this study, questions on mold spots, damp stains, window pane condensation and cleaning frequency are based on child's bedroom. This could lead to some miss-classification of parents' exposure. However, we still found strong associations between dampness and parents' rhinitis symptoms. Usually, Chinese homes are not very big and bedrooms are close to each other. Thus, dampness and other impaired factors could influence the whole family members.

While statistical models can affect results, consistent results have been obtained from different tests, and good agreement was found between multiple logistic regression models and logistic regression models using stepwise method.

Previous studies have shown that the most frequent sensitization sources are both perennial allergens (house dust mites) and seasonal allergens (spring pollens and autumn pollens) [42]. The peak seasons for pollen in Chongqing is in March in spring and in autumn from September to October. Spring pollens accounting for 65.8% of the annual pollen spreading, and the main airborne pollens are from pinus, moraceae and cupressaceae [43]. Allergen testing allergy patients shows that the main pollen allergen is in Autumn in Chongqing [43]. Another study in Chongqing showed that there were two peaks of AR, one in early-spring and another mid-autumn [44]. Our questionnaire study was performed from December 2010 to April 2011, and parents were asked about their rhinitis symptoms in the last three months (current rhinitis) which was about the time of the pollen and infection peak season. The prevalence of current rhinitis in our study is comparable with another cross-sectional, population-based study in Chongqing from 2008 [44] using the ISAAC questionnaire [45] for rhinitis which showed a prevalence of 32.3% self-reported AR in the last 12 months. The majority of subjects who had a history of allergic rhinitis reported current rhinitis in our study indicating that they could exposed to the allergens during the study recall period.

We found that current smokers reported less history of allergic asthma. This negative association has been shown in studies about allergic sensitization and smoking habit [46], [47]. Selection could be the reason for this finding, as the awareness of smoking is harmful for asthmatic people, those with asthma choose to avoid smoking. However, we have found that passive smoking (spouses' current smoking) was a risk factor for non-smoking females' current rhinitis. Our finding that history of allergic asthma was associated with more reports of rhinitis symptoms is in line with other studies [7], [12].

Associations between tobacco smoke odor and current rhinitis as well as history of allergic rhinitis were found in our study. Moreover, stuffy odor, unpleasant odor and the perception of dry air were all significantly associated with current rhinitis. Mold odor was not a risk factor in our study, however, it was the largest risk observed in one review article on dampness and molds with rhinitis risk [48]. Perception of strong odors has been shown to be a predisposing factor for NAR patients [49]. These perceptions could be indicators of a lack of ventilation as well as emissions from indoor sources.

A noteworthy finding in our study is that people living near a main road or highway reported more rhinitis symptoms. One Chinese study has shown that living or working place near the traffic artery had 1.94-fold increased risk for development of NAR compared with AR [49]. One Swedish study found that living within 100 m from a road with high traffic intensity was associated with a higher prevalence of allergic rhinitis for adults, but not with rhinitis triggered by non-allergic factors [50]. An Italian study in adults found that outdoor traffic related NO_2_ exposure was associated with increased prevalence of allergic rhinitis [51]. Traffic pollutions may potentiate allergic reactions in different ways [52]: firstly, by attaching to the surface of e.g. pollen grains, air pollutants can change their morphology and enhance allergenic potential; secondly, by inducing inflammation; thirdly, diesel exhaust emissions can have an adjuvant effects in atopic subjects. We did not measure outdoor pollutants, but one study on the air pollutants at the highway toll gates in Chongqing showed that the indoor and outdoor average concentrations of CO, NO_2_ and PM_10_ exceeded indoor air quality standards [53]. Moreover, a recent study on air fine particles pollution in Chongqing showed that PM_2.5_ exposure in the city came mainly from vehicle emission [54].

The present study confirms the findings of many previous studies that indoor dampness and mold are risk factors for rhinitis and asthma. Excess moisture in buildings becomes a critical factor for mold (fungal) proliferation in nutrient-rich environments, as well as for house Dust Mites. As a result, building occupants may be exposed to increased levels of microbial agents. Consistent positive associations between dampness and multiple allergic, respiratory effects have been shown [55]. The risk of rhinitis is significantly increased in relation to home dampness and mold exposures [48]. The risk of current asthma, current asthma symptoms, allergic rhinitis, and atopic dermatitis was higher in homes with self-reported dampness in the past year among 18–25 years Finish students [18]. Another Finish study found that self-reported mold in homes was associated with rhinitis among adults [56]. One American study found that water stains was associated with students' nasal symptoms in college buildings [57]. The association between dampness in dwellings and wheeze, asthma among adults has been well studied [58]. Even if the mechanisms of the effects of dampness on health are unknown, there is sufficient evidence to find out and remediate the reasons for the dampness problem and take preventive measures against dampness in buildings [48], [58]–[60]. The result of our study could guide practical prevention and remediation for Chongqing, as it has a humid subtropical monsoon climate. An annual outdoor average relative humidity was around 78% [44].

This study showed that the presence of cockroaches was associated with an elevated risk of rhinitis symptoms. One Chinese study has found that cockroach allergens were detected in 93% of the household samples [61]. Exposure to cockroaches was also found to be associated with wheeze in Chinese ninth grade students [62]. 25.7% of the patients with allergic rhinitis and asthma had positive cockroach sensitization in mainland China in one Chinese study [63].

We found that pet keeping was associated with an elevated risk of having rhinitis symptoms, this is in agreement with another study reported that the prevalence of rhinitis was more common in families with animals than in those without [13]. The association between pet allergens (such as allergens from cats and dogs) and allergic symptoms has been widely discussed in epidemiological studies. Cats and dogs were the most common pets in our study (5.1% of the parents reported having cats and 6.9% reported having dogs). In rural areas in China, cat and dog epithelium in rural areas were the most common allergens [64].

Our study found that redecoration and new furniture at home were risk factors for parents' rhinitis symptoms. Redecoration and new furniture could be a source of chemical emissions. Factor analysis showed that these two exposures were associated with each other. Buying new furniture is usually done the same time as redecoration in China. Although this study did not measure indoor pollutants, chemical emissions, such as formaldehyde and volatile organic compounds (VOC) from furniture and decorating process has been studied [65]–[67]. We have not found any study on the association between renovation, new furniture or new materials and rhinitis. However, the association between renovation or new materials and asthma-related and lower respiratory symptoms have been studied: recent painting was associated with wheezing in the last 12 months and any allergy in Russia schoolchildren [66]; new wall covering and new furniture were associated with any allergy [66]; new synthetic carpet was associated with current asthma and any allergy [66]. The presence of particleboard (formaldehyde-emitting materials) was associated with current asthma and any allergy [66]. Plastic-containing or other materials (possible VOC emitting materials), such as new linoleum floor, PVC flooring, textile wall covering, synthetic bedding, were found to be associated with history of asthma, asthma-related or bronchial obstruction in children [66], [68], [69]. One home study found exposed to higher formaldehyde levels in the bedroom were associated with atopy and increasing severity of sensitization among children [70]. Expose to high level of formaldehyde increased nasal mucosal hypersensitivity to histamine in medical students during cadaver dissection [71]. One toxicological chamber study found provocation with formaldehyde at a dose of 0.5 mg/m3 over 2 hours caused transient rhinitis symptoms in both patients with skin sensitization and healthy subjects [72]. One French study found that high concentrations of VOC in homes were associated with an increasing prevalence of asthma and rhinitis in adults [73].

Cleaning every day and frequently put bedding to sunshine were protective factors for rhinitis in our study. Factor analyses showed that cleaning every day and frequently put bedding to sunshine were associated with each other as one factor. Frequently cleaning and putting bedding to sunshine could be associated with reduced risk of impaired indoor environment. We found no other study about the association between the frequency of cleaning, putting bedding to sunshine and rhinitis symptoms among adults in the home environment. However, an intervention study found that comprehensive cleaning reduced the airborne dust and also reduced nonsmokers' mucosal symptoms and nasal congestion in offices [74]. House dust mites are commonly found in beds. One Chinese survey found that the most common allergen was house dust mites in urban homes [64]. Another Chinese study found that house dust mite allergen levels were detectable in 99% of the bedding samples [61]. Frequently putting bedding to sunshine could reduce house dust mite allergens.

## Conclusions

Factors in the home environment, especially living near a main road or highway, redecoration, new furniture, mold spots, water damage, window pane condensation in winter, having pets may increase the risk of having current rhinitis and history of allergic rhinitis in Chinese residents. The results of this study indicate a need to reduce dampness and indoor mold growth, use low emission furniture and paints in homes and encourage frequent cleaning and putting bedding to sunshine to decrease occupants' risk of having rhinitis symptoms in indoor environment.
